# AARP Age-Friendly community designation and neighborhood resources to support healthy aging nationwide, 2012–2017

**DOI:** 10.1016/j.healthplace.2026.103613

**Published:** 2026-01-20

**Authors:** Alexandra Eastus, Yvonne L. Michael, Stephen Dickinson, Stephen Francisco, Steven Melly, Jana Hirsch

**Affiliations:** a Department of Health Management and Policy, Dornsife School of Public Health, Drexel University, Nesbitt Hall, 3215 Market St., Philadelphia, PA, 19104, USA; b Department of Epidemiology and Biostatistics, Dornsife School of Public Health, Drexel University, Nesbitt Hall, 3215 Market St., Philadelphia, PA, 19104, USA; c Urban Health Collaborative, Dornsife School of Public Health, Drexel University, Nesbitt Hall, 3215 Market St., Philadelphia, PA, 19104, USA

## Abstract

**Objective:**

To examine the association between city-level Age-Friendly designation and neighborhood resources that support healthy aging at the census tract-level.

**Methods:**

We quantified the density of neighborhood resources in census tracts that support healthy aging using the National Establishment Time Series (NETS). We identified cities designated as American Association of Retired Persons (AARP) Network of Age-Friendly States and Communities Members. Using a propensity score matched regression, we estimated the association between AARP Age-Friendly membership designation and the density of neighborhood resources that support healthy aging within census tracts.

**Results:**

Our matched sample consisted of 134,031 census tract-years between 2012 and 2017. Our findings suggest that census tracts located in Age-Friendly designated cities are associated with greater densities of neighborhood resources that support healthy aging overall, as well as for business establishments that facilitate aging in place (e.g., healthcare facilities).

**Conclusion:**

These findings suggest a meaningful association between local Age-Friendly designation and the availability of resources that can support aging in place. Although we did not directly measure health outcomes, neighborhoods with greater resource density are likely to experience multiple health benefits, including improved quality of life for older adults.

## 1. Introduction

Global population demographics are changing; people are living longer lives, shifting population age structures (Wilmoth et al., 2023). The number of people over the age of 65 is expected to double by 2050 globally (Wilmoth et al., 2023). While success in public health campaigns and medical innovations have contributed to increases in lifespan, not everyone who lives far into adulthood experiences good health and a comfortable standard of living. Although genetic and predisposing factors contribute to diseases in adulthood (e.g., dementia or cardiovascular disease), environmental risk factors and other social determinants of health greatly influence the health and well-being of individuals (Brewster et al., 2019; Bonaccorsi et al., 2020). Despite global increases in life expectancy before the COVID-19 pandemic – gains that have inconsistently returned to pre-pandemic levels – significant disparities in aging persist both between and within populations (Forrester et al., 2020; Abeliansky et al., 2020; Woolf, 2023). Further, there are inequities in healthy aging, or the ability to age while maintaining good physical, mental, and social health (Belsky and Baccarelli, 2023; Tipirneni et al., 2019).

Typically aging research has focused on the impact of individual-level risk factors on healthy aging (Chakravarty et al., 2012; Castruita et al., 2022; Atallah et al., 2018). For example, individual lifestyle factors directly impact the risk of chronic and metabolic disease development, cognitive functioning, functional independence, and overall physical and mental health (Atallah et al., 2018). Specifically, individuals who consume nutrient-dense diets, maintain moderate physical activity levels, limit alcohol intake, and are otherwise free of pain and illness are more likely to experience healthy aging (Castruita et al., 2022). At the individual level, risk and protective factors for health are also heavily influenced by upstream determinants, discriminatory policies, and systems of oppression (Ray et al., 2023). Despite the importance of upstream determinants on health outcomes, many crucial research gaps persist regarding cognitive health and aging risk factors at the neighborhood and community levels. In particular, limited research has quantified how social policies that promote healthy aging impact the built environment, which limits knowledge of potentially policy-relevant factors (Hirsch et al., 2022). Previous research has investigated the role that environments play in cognitive function throughout the life course (Bonaccorsi et al., 2020; Durfey et al., 2019; Lei and Beach, 2023; Yu et al., 2023). A few studies have measured how neighborhood environments and the features of neighborhoods (e.g., types of business establishments) influence healthy aging (Bonaccorsi et al., 2020; Lei and Beach, 2023; Yu et al., 2023). These findings show how social advantages or disadvantages impact the composition of neighborhoods and the accessibility of resources in older adulthood. In response to the rise of aging populations, research and policy have increasingly focused on mechanisms to enhance access to resources that support healthy aging, including the “Age-Friendly” movement.

In response to the global aging population, the World Health Organization created the global Age-Friendly movement to advocate for the advancement of accessible communities for all– with a focus on ensuring access for older adults (Wilmoth et al., 2023). A key aspect of this movement is designing the built environment to promote the social inclusion of older adults, with the idea that communities that support aging also benefit people of all ages (Salmistu and Kotval, 2023). This global movement has aided in the development of a network of Age-Friendly states and communities in the United States, led by the American Association of Retired Persons (AARP) (AARP, 2025). AARP’s Age-Friendly Network is a locally driven national initiative that works toward improving livability in cities, counties, and states throughout the United States (AARP, 2025). Supplementary Figs. 1–3 showcases the cities, counties, and states that have opted in to the network as of 2022. As part of this network, member cities, counties, and states can share technical assistance and expertise with each other. While belonging to the Age-Friendly network does not mean that AARP has endorsed the location as Age-Friendly (AARP, 2025), membership in the network indicates that the elected leadership has opted-in to work towards improving the livability of the community for all ages (AARP, 2025). As part of this commitment, places designated as Age-Friendly are required to conduct a community needs assessment to assess and respond to the needs of the aging population (AARP, 2025).

To our knowledge, no studies have assessed the relationship between AARP’s Age-Friendly designation and the availability of neighborhood resources to support healthy aging. This is a significant gap, given that 786 communities, cities, counties, and states, are part of the Age-Friendly network, encompassing 100 million residents (AARP, 2025). In these designated communities, routine assessment and political commitment may help advance infrastructure that supports healthy aging, with benefits extending to people of all ages. To address this gap, we conducted a propensity score-matched analysis to examine the association between AARP Age-Friendly designation and the density of neighborhood resources that support healthy aging. We hypothesized that census tracts in designated cities will have higher densities of these resources compared to tracts in non-designated cities.

## 2. Methods

### 2.1. Data and study population

We linked geo-identified data from the American Community Survey (ACS), National Establishment Time Series (NETS), and AARP Age-Friendly Membership data to construct a longitudinal census tract-level dataset covering 2012–2017 (AARP, 2025; Bureau USC, 2012; Hirsch et al., 2021; Kaufman et al., 2015). This study period was selected because 2012 was the year that the AARP established the Age-Friendly Network. The 2019 NETS dataset includes annual micro-level records for over 72 million unique business establishments across the U.S., including industry classification, employment, and business type.

All contiguous U.S. census tracts were eligible. We excluded tracts with populations less than 50, those designated as special-use areas (e.g., airports, parks), or with missing covariate data. we excluded census tract-year observations that did not have complete observations (n = 580,166), resulting in a pre-matched analytic sample of 430,224 census tract-year tract-years from 2012 to 2017.

### 2.2. Exposure: AARP age-friendly designation

The primary exposure was city-level AARP Age-Friendly designation. Although designations can occur at the county and state levels, we focused on cities because local governments typically have more direct authority over zoning, land use, transportation, and service provision, factors that strongly influence the neighborhood environment and location of businesses that support healthy aging.

**Fig. 1.**
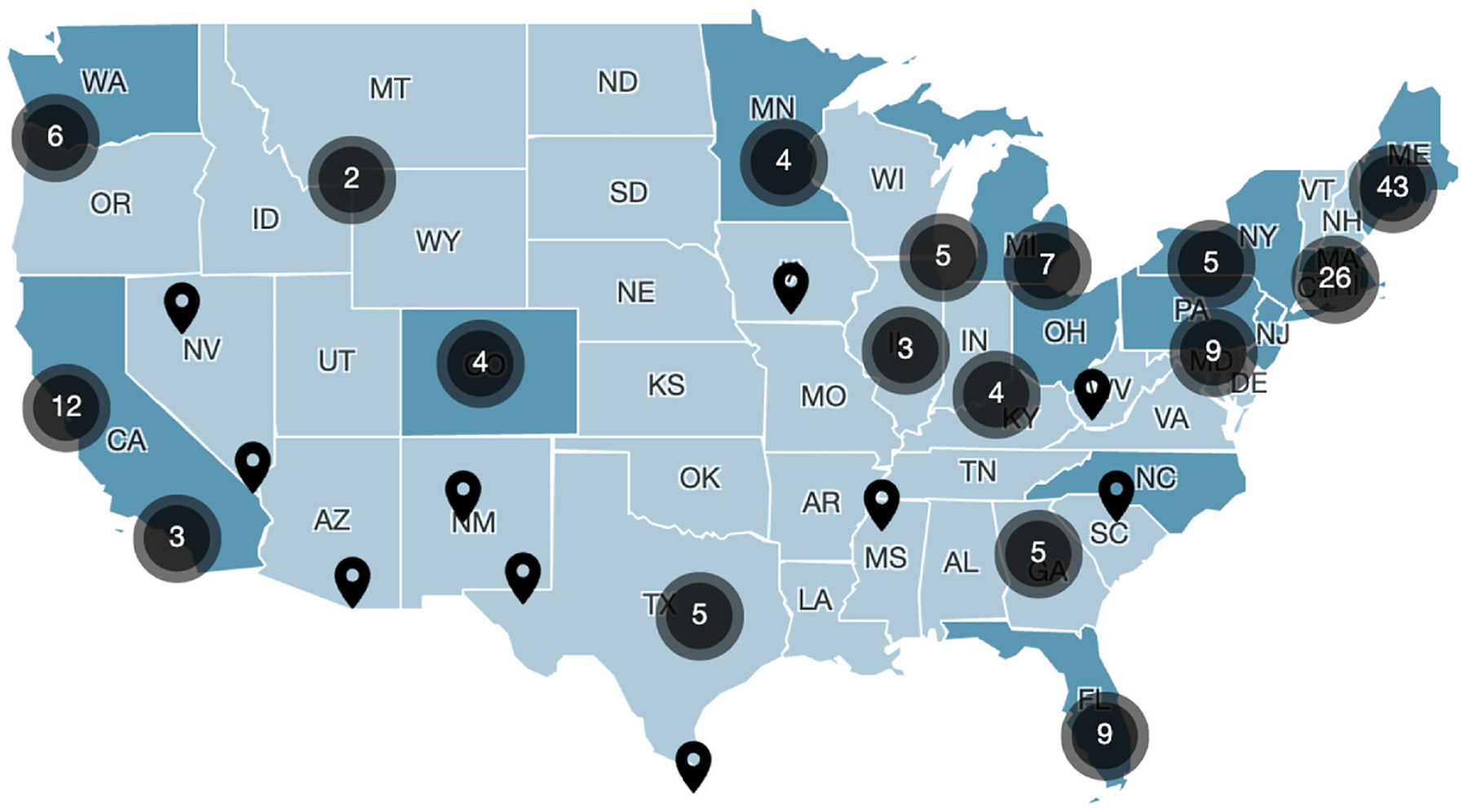
Distribution of AARP Age-Friendly cities (2012–2017). Note: This map reflects Age-Friendly **city/town** designations, which were used in the analytic sample. Although some states and counties also participate in the AARP Age-Friendly Network, county- and state-level designations were not included in the analysis. States shaded in dark blue indicate statewide participation in the network during the study period; these are shown for contextual reference only.Icons with numbers represent the count of designated cities/towns that are near each other. *Source:* AARP. “AARP Network of Age-Friendly States and Communities.” Accessed in 2025, from https://www.aarp.org/livable-communities/network-age-friendly-communities/.

We manually collected designation status and year of designation from the AARP Age-Friendly Network (AARP, 2025). Fig. 1 illustrates the geographical distribution of AARP Age-Friendly cities during the study period. Since census tracts are not necessarily nested within city (incorporated places) boundaries, using 2018 TIGER/Line shapefiles and ArcGIS, we spatially joined designated cities to census tracts (using 2010 census designations) to calculate the proportion of each tract's area which overlapped with a designated geography. Tracts with greater than or equal to 50 % overlap were coded as designated. A binary variable was created indicating Age-Friendly membership (1 = census tract falls in designated city, 0 = census tract does not fall within designated city). All census tracts were also categorized as “ever treated”, indicating that they resided in a city that opted into the AARP Age-Friendly Network between 2012 and 2017 or “never treated”, indicating that the census tracts did not reside in an AARP Age-Friendly city.

## 3. Outcome: neighborhood resources for healthy aging

We used NETS data and a classification framework developed by the MESA Neighborhoods and Aging team to quantify tract-level business establishments relevant to healthy aging. This framework builds on prior work from the Retail Environment and Cardiovascular Disease Study (RECVD) (Hirsch et al., 2022) and aligns with the concept of cognability, which captures neighborhoods features that shape physical, cognitive, and social functioning in older adulthood (Finlay et al., 2022). This study integrates measures of neighborhood supports for healthy aging into the existing Multi-Ethnic Study of Atherosclerosis (MESA), leveraging its comprehensive longitudinal sociodemographic, behavioral, and health data. We incorporated both survey-based and Geographic Information Systems (GIS) measures to capture neighborhood factors that influence physical function, cognitive function, and the ability to age in place. Survey data included assessments of food environments, safety, and social cohesion, as well as items relevant for mobility, cognition, and aging in place. GIS-based measures built on prior MESA/RECVD classifications and were aligned with cognability. Through literature review, conceptual modeling, and harmonization of existing frameworks, we developed a comprehensive schema of neighborhood resources to advance the understanding of how place shapes healthy aging.

Establishments were categorized into three domains:

1. Physical Domain: Businesses supporting physical activity and well-being (e.g., fitness centers, alternative medicine providers, and recreational facilities).
2. Cognitive Domain: Includes establishments supporting intellectual and social engagement, such as museums, libraries, religious organizations, and cultural or hobby-based institutions.
3. Aging in Place Domain: Includes businesses that enable older adults to live independently, including food stores, healthcare providers, social services, financial institutions, and senior living facilities.

An aggregate measure combining all three domains was used as the primary outcome of interest. We decided to create an aggregate measure because of the interconnected nature of the different business domains as well as the hypothesis that all business establishments may directly and indirectly impact health. In secondary analyses, we evaluated the relationship between Age-Friendly city-designation and the density of neighborhood resources that support healthy aging, specific to establishments in each domain (physical, cognitive, and aging in place). For each tract-year, we calculated counts and densities (per km (Brewster et al., 2019)) of unique business establishments in each category.

### Covariates.

We selected tract-level sociodemographic variables hypothesized to confound the relationship between Age-Friendly designation (exposure) and neighborhood resource density (outcome) (Supplementary Figure 4). All covariates were derived from ACS 5-year estimates, 2012–2017 (Bureau USC, 2012). The ACS is a 1 % sample of the U.S. noninstitutionalized population that collects data on social, economic, housing, and demographic characteristics (Bureau USC, 2012). The 5-year ACS data include pooled estimates (e.g., 2010–2014), which can be used to quantify annual estimates using the mid-point year (e.g., 2012). Using 5-year ACS estimates, we adjusted for census-tract level median household income, educational attainment, racial/ethnic composition, population density, and the population of adults over the age of 65.

#### 3.1. Statistical analysis

We conducted a series of descriptive and inferential analyses to evaluate the relationship between AARP Age-Friendly designation and neighborhood resource density. First, we calculated frequencies, means, and standard errors for all variables, and compared ever-treated and never-treated census tracts before matching. We also compared census tracts in the included analytic sample to those excluded due to missing data or special-use designation to assess potential for selection bias. To estimate the effect of city Age-Friendly designation on neighborhood resource resources, we used one-to-one nearest neighbor propensity score matching (Austin, 2010). Designated census tracts were matched to control tracts with similar characteristics, and treatment effects were estimated using generalized linear models.

Propensity scores were estimated based on census tract-level confounding factors, and matching was used to create comparable treatment (e.g., Age-Friendly designated census tracts) and control groups (e.g., no designation) (Matthay and Glymour, 2022). Matching was performed with replacement to allow control tracts to be matched with multiple treated tracts, which can improve the quality of the match and reduce bias in cases of limited overlap between control and treated units. A caliper of 0.1 on the propensity score scale was used to restrict matches to control units with a specified distance of the treated unit's score, thereby minimizing the risk of poor matches and reducing residual confounding. Treated tracts were matched to the control tract with the most similar propensity score, increasing the exchangeability between treated and control census tracts based on sociodemographic characteristics.

To assess the adequacy of the propensity score match, we examined the degree of common support between the treated using Balance and Love plots. Covariate balance was evaluated using standardized mean differences (SMDs), with an SMD of less than 0.1 indicating acceptable balance between the groups. Love plots and balance tables demonstrating common support and covariate balance between the treatment and control groups are presented in Supplementary Figures 5–6.

Treatment effects were estimated using linear regression models, with coefficients representing the average treatment effect on the treated (ATT) and 95 % confidence intervals. We used RStudio Version 2022.12.0 + 353 (2022.12.0 + 353) to compile the data and the *MatchIt* package to perform analyses (Ho et al., 2018).

#### 3.2. Sensitivity analysis

To assess the robustness of our findings, we estimated standard multivariable linear regression models on the propensity score-matched sample, adjusting for the same set of covariates used to generate the propensity score. These models provide an alternative specification to confirm that the results were not driven by the matching procedure. Importantly, this step also helps evaluate the potential for residual confounding, by testing whether the adjustment for observed covariates produces results consistent with those obtained from the matched analysis.

## 4. Results

The propensity score matching procedure yielded 78,998 treated and 55,033 matched control units (census tracts-years), with some controls reused across multiple treated units. Control units not selected in the matching process (n = 295,881) were excluded, and an additional 312 were discarded due to caliper restriction. The effective sample size of matched controls was 19,677, reflecting the reuse of control units.

Table 1 presents pre-matching descriptive statistics comparing census tracts ever treated (e.g., located in cities that opted into the AARP Age-Friendly Network; n = 13,171) to never treated tracts (n = 58,536). Before matching, ever-treated tracts were more densely populated (mean population density = 6276 vs. 1532 people per square mile, p < 0.001), had slightly lower median income, and were less likely to have non-Hispanic White residents, and somewhat more likely to have non-Hispanic Black residents.

After matching, the analytic sample included 13,171 census tracts in designated cities and 12,520 in non-designated cities (Table 2). Matching substantially improved covariate balance, with median values for population size, educational attainment, and household income similar across groups (Table 2). Small differences remained: designated city tracts had slightly higher population density (median = 6276 vs. 5494 people per square mile) and a modestly higher share of non-Hispanic White residents (median = 0.48 vs. 0.43), while distributions of other racial-ethnic groups were comparable. These results suggest the groups were well balanced overall, with only minor differences in urbanicity and racial composition (Table 2).

In the aggregate model, census tracts in cities with AARP Age-Friendly designation had, on average, 79.68 more neighborhood resources that support healthy aging compared to matched non-designated tracts (95 % CI: 68.60, 90.81; *p* < 0.001; Table 3). In secondary analyses, Age-Friendly designation was consistently linked to higher availability of supportive neighborhood establishments across multiple dimensions including Physical (β = 1.30; 95 % CI: 1.15, 1.45), Cognitive (β = 4.19; 95 % CI: 3.83, 4.55), and Aging in Place (β = 16.17; 95 % CI: 14.20, 16.60) domains (Table 3).

In sensitivity models using multivariable regression on the matched sample, census tracts in cities with AARP Age-Friendly designation had, on average, 103.78 more neighborhood resources that support healthy aging compared to matched non-designated tracts (95 % CI: 91.84, 115.71; *p* < 0.001). Across specific domains, Age-Friendly designation was consistently associated with higher availability of supportive neighborhood resources including Physical (β = 1.26; 95 % CI: 1.11, 1.41), Cognitive (β = 3.62; 95 % CI: 3.23, 4.01), and Aging in Place (β = 17.30; 95 % CI: 15.09, 19.51) domains (Table 3).

## 5. Discussion

In this study, we found that census tracts located in cities with AARP Age-Friendly designation had a significantly higher density of neighborhood resources that support healthy aging, even after accounting for sociodemographic characteristics such as income, education, and race/ethnicity. These findings suggest a meaningful association between local Age-Friendly designation and the availability of resources that can support aging in place.

These results complement a growing body of research highlighting the role of neighborhood environments in shaping health outcomes across the life course, including in older adulthood. Prior studies have shown that adverse neighborhood conditions are associated with increased risk of chronic disease, functional decline, and premature mortality (Diez et al., 2010). More recently, focus has shifted to the potential for neighborhood environments to promote resilience and well-being in older age, emphasizing the importance of access to health-supporting infrastructure such as transportation, healthcare services, social spaces, and walkable environments (Diez Roux and Mair, 2010; Durfey et al., 2019; Yu et al., 2023; Cheung, 2022; Martin et al., 2021; Yang and Stone, 2024). Our findings expand on this work by identifying a potential policy-relevant factor, AARP Age-Friendly designation, that may be linked to the presence of neighborhood resources and amenities. In particular, this study seeks to examine the implementation of a policy that is intended to provide education to elected officials and residents, as well as provide localities with the necessary resources (research findings, best practices) to become more age-friendly (AARP, 2025).

One plausible explanation for our findings is that communities seeking Age-Friendly designation may already possess the political will and organizational capacity to invest in age-supportive changes. This may reflect both a selection effect, in which communities already committed to Age-Friendly planning pursue designation, and a catalytic effect, in which designation reinforces local investment in supportive infrastructure. In a comparison of unmatched designated and non-designated census tracts, we found that census tracts in designated cities tended to have greater racial and ethnic diversity, slightly lower educational attainment, lower median household income, and lower proportion of older adults (aged 65 and older) (Table 1). These patterns suggest that AARP Age-Friendly designation may not simply reinforce existing advantage. Instead, cities with more diverse and socioeconomically mixed populations may view the Age-Friendly designation as a tool to attract investment, foster public-private partnerships, or raise awareness to address community needs and gaps in resources (Salmistu and Kotval, 2023). Importantly, the AARP Age-Friendly Network requires jurisdictions to commit to ongoing planning and assessment, which can catalyze investment in built environments, public services, and collaborative and interdisciplinary partnerships that prioritize aging populations (Chattopadhyay, 2024).

This study builds on prior and ongoing research by explicitly evaluating the implementation of AARP Age-Friendly policies and how city-designation in census tracts impacts neighborhood resources. While we cannot establish temporal order, our findings suggest that Age-Friendly designation is associated with neighborhoods that are better resourced to support aging. These resources, including food stores, healthcare services, transportation options, and social gathering places, are essential for maintaining independence, mobility, and social engagement for older adults. Our findings highlight the potential of Age-Friendly community frameworks as a tool to foster more supportive environments for aging populations, which has many implications for policymakers, urban planners, and researchers (Salmistu and Kotval, 2023). As the aging population continues to increase, collaboration across sectors will be necessary to ensure built environments accommodate aging in place, with attention to differences between rural and urban contexts. Future research should examine whether AARP Age-Friendly designation is associated with downstream health outcomes, such as chronic disease management, and qualitatively assess the motivations of policymakers to opt into the Age-Friendly Network.

## 6. Strengths and limitations

This study has important strengths. First, to our knowledge, it is the first to evaluate the relation between AARP Age-Friendly designation and neighborhood resources that support healthy aging. Second, we employed a quasi-experimental design using propensity score matching to construct a comparison group of non-designated census tracts that were otherwise similar to designated tracts on observed characteristics. This approach enhances internal validity by minimizing the risk of residual confounding. Third, we used a large national sample with detailed census tract-level data on neighborhood resources, measured objectively over multiple years and not subject to bias by resident self-report. Finally, our sensitivity analyses found similar effect estimates in both direction and magnitude to the primary propensity score matched analysis, supporting the robustness of our findings.

Several limitations should be noted. First, our analytic scope focuses exclusively on city-level AARP Age-Friendly designations. County- and state-level designations were not included, as our objective was to assess neighborhood-level resources associated specifically with municipal Age-Friendly initiatives. Accordingly, only census tracts located within the boundaries of designated cities were classified as treated; tracts in counties with Age-Friendly designations but not located within designated municipalities were not considered Age-Friendly. This operational definition may introduce misclassification, as tracts do not perfectly align with city boundaries, which would likely bias estimates toward the null. Second, 2267 census tracts were excluded due to matching constraints, which may introduce selection bias, although observed differences between included and excluded tracts were small and consistent with data missing at random. Third, despite propensity score matching, slight imbalances remained in density and racial composition, potentially introducing residual confounding. However, results from multivariable models suggest this bias is minimal. Fourth, because our unit of analysis was census tract-year, findings should be interpreted at that level. Finally, we cannot establish temporal order; the study did not use pre-post comparisons or other quasi-experimental designs. Future research should evaluate whether Age-Friendly designation causally increases resource density using approaches such as staggered difference-in-differences.

## 7. Conclusion

This study contributes novel evidence to the growing literature on Age-Friendly policy frameworks by empirically evaluating their neighborhood-level correlates using a rigorous matching approach. We found that Age-Friendly designated cities have more neighborhood resources to promote healthy aging than non-designated cities. Our findings support the potential of Age-Friendly community frameworks as a tool to foster more supportive environments for aging populations.

## Supplementary Material

1

## Figures and Tables

**Table 1:** Baseline descriptive statistics of census tracts (pre-matching, 2012) by age-friendly membership status.

	City Membership Status	
	Ever City Member	Never City Member
	n = 13,171	n = 58,536
**Total population**		
Median (IQR)^a^	3928.00 (2842.00, 5234.00)	4100.00 (2948.50, 5466.00)
**Population Density (per Square Mile)**		
Median (IQR)^a^	6276.20 (3367.14, 14,810.11)	1532.22 (193.61, 4117.48)
**Number of People 65 years and older (in census tract)**		
Median (IQR)^a^	465.00 (295.00, 693.00)	598.00 (396.00, 847.00)
*Race & Ethnicity (% of Census Tract Population)*		
**Hispanic**		
Median (IQR)^a^	0.14 (0.05, 0.35)	0.06 (0.02, 0.16)
**White, non-Hispanic**		
Median (IQR)^a^	0.48 (0.14, 0.74)	0.77 (0.50, 0.91)
**Black, non-Hispanic**		
Median (IQR)^a^	0.07 (0.02, 0.25)	0.03 (0.01, 0.13)
**Asian, non-Hispanic**		
Median (IQR)^a^	0.02 (0.01, 0.03)	0.01 (0.01, 0.03)
**Two or More Races, Non-Hispanic**		
Median (IQR)^a^	0.02 (0.01, 0.03)	0.01 (0.01, 0.03)
*Educational Attainment (% of Census Tract Population)*		
**High School Diploma GED or alternative**		
Median (IQR)^a^	0.23 (0.15, 0.31)	0.26 (0.18, 0.34)
*Census Tract Median Income ($)*		
**Median Household Income** ^b^		
Median (IQR)^a^	53,642.00 (36,744.00, 77,723.00)	58,882.00 (42,357.00, 82,905.00)

Note: Race/ethnicity & Educational Attainment median values represent proportions of the population.

a Interquartile Range (IQR).

b Median household income in the past 12 months. Note: Income here represents income in the past 12 months in 2017 inflation-adjusted dollars.

**Table 2:** Baseline descriptive statistics of census tracts (matched), 2012 by age-friendly membership status.

	City Membership Status	
	City Member	Not City Member
	n = 13,171	n = 12,520
**Total population**		
Median (IQR)^a^	3928.00 (2842.00, 5234.00)	4046.50 (2879.50, 5299.00)
**Population Density (per Square Mile)**		
Median (IQR)^a^	6276.20 (3367.14, 14,810.11)	5493.78 (2403.40, 13,198.12)
**Number of People 65 years and older (in census tract)**		
Median (IQR)^a^	463.00 (295.00, 693.00)	445.00 (287.00, 654.00)
*Race & Ethnicity (% of Census Tract Population)*		
**Hispanic**		
Median (IQR)^a^	0.14 (0.05, 0.35)	0.14 (0.05, 0.37)
**White, non-Hispanic**		
Median (IQR)^a^	0.48 (0.14, 0.74)	0.43 (0.16, 0.69)
**Black, non-Hispanic**		
Median (IQR)^a^	0.07 (0.02, 0.25)	0.07 (0.02, 0.25)
**Asian, non-Hispanic**		
Median (IQR)^a^	0.03 (0.01, 0.10)	0.03 (0.01, 0.10)
**Two or More Races, Non-Hispanic**		
Median (IQR)^a^	0.02 (0.01, 0.03)	0.02 (0.01, 0.03)
*Educational Attainment (% of Census Tract Population)*		
**High School Diploma GED or alternative**		
Median (IQR)^a^	0.25 (0.17, 0.32)	0.26 (0.18, 0.33)
*Census Tract Median Income ($)*		
**Median Household Income** ^b^		
Median (IQR)^a^	48,456.00 (33,244.00, 69,500.00)	49,818.00 (35,446.00, 70,519.50)

Note: Race/ethnicity & Educational Attainment median values represent proportions of the population.

a Interquartile Range (IQR).

b Median household income in the past 12 months. Note: Income here represents income in the past 12 months in 2010 inflation-adjusted dollars.

**Table 3:** Matched adjusted regression results (1).

	Matched Regression			
	Aggregate Model (2) [95 % CI]	Physical Domain (2) [95 % CI]	Cognitive Domain (2) [95 % CI]	Aging in Place Domain (2) [95 % CI]
**Adjusted Regression**				
Density of neighborhood resources that support healthy aging per square mile	79.68 [68.60, 90.80] ***	1.30 [1.15, 1.45] ***	4.19 [3.83, 4.55] ***	16.17 [14.20, 16.60] ***

Significance codes:

* p < 0.05,

** p < 0.01,

*** p < 0.001.

(1) Matched cases and controls derived from propensity score matching.

(2) Descriptions of each of the models: Physical Domain: Businesses supporting physical activity and well-being (e.g., fitness centers, alternative medicine providers, and recreational facilities); Cognitive Domain: Includes establishments supporting intellectual and social engagement, such as museums, libraries, religious organizations, and cultural or hobby-based institutions; Aging in Place Domain: Includes businesses that enable older adults to live independently, including food stores, healthcare providers, social services, financial institutions, and senior living facilities; Aggregate Model: Combination of all business establishments represented in the Physical, Cognitive, and Aging in Place Domains.

## Data Availability

Data will be made available on request.

## Appendix A. Supplementary data

Supplementary data to this article can be found online at https://doi.org/10.1016/j.healthplace.2026.103613.
