# Supplementary material for: AARP Age-Friendly community designation and neighborhood resources to support healthy aging nationwide, 2012–2017
Source: Health Place. Author manuscript; Available in PMC 2026 Jun 30. (PMC13318090; doi:10.1016/j.healthplace.2026.103613)

**APPENDIX**

**Supplementary Figure 1**: Distribution of AARP age-friendly cities.


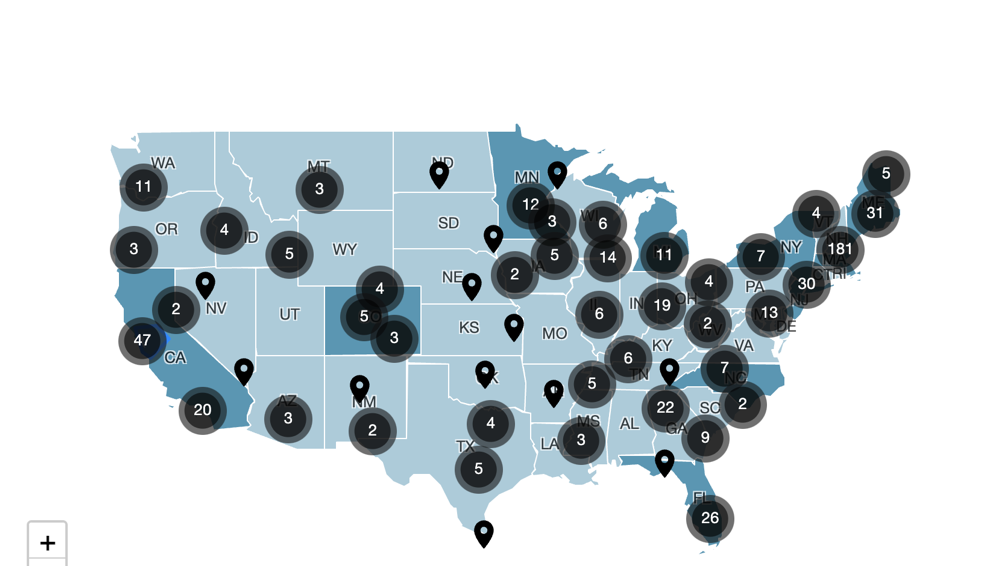


**Supplementary Figure 2**: Distribution of AARP age-friendly counties.


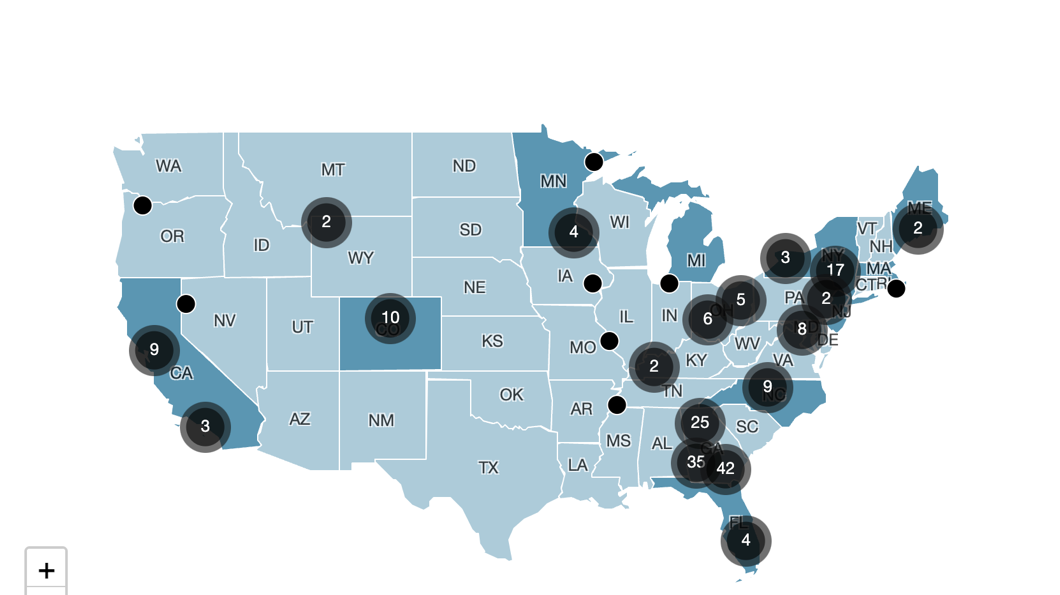


**Supplementary Figure 3**: Distribution of AARP age-friendly states.


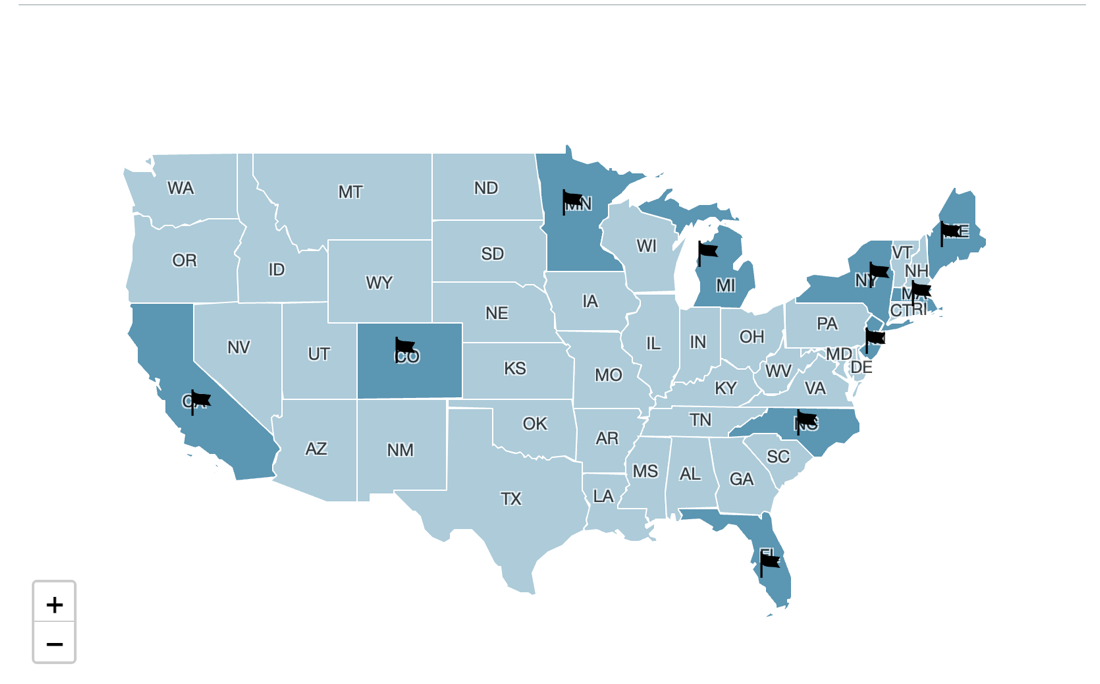


**Supplementary Figure 4**: Directed Acyclic Graph for Age-Friendly Designation and Neighborhood Resources


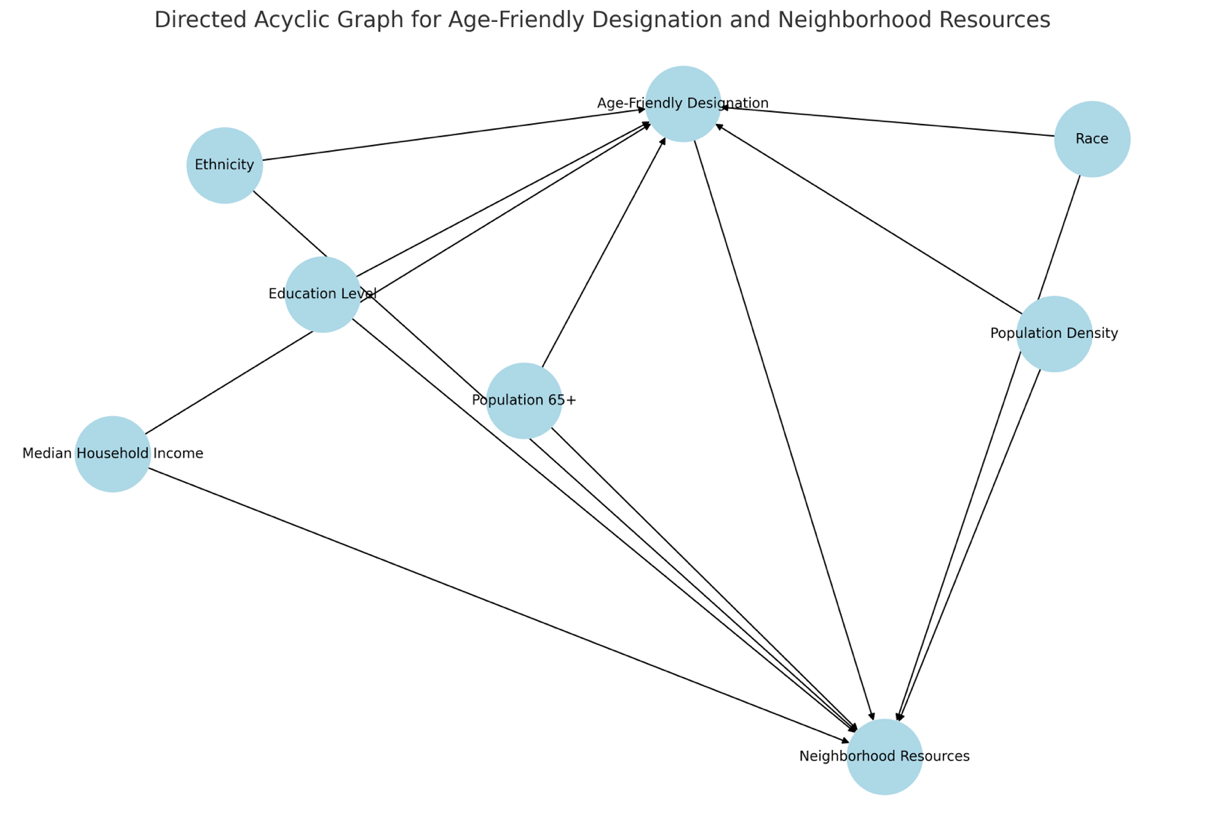


**Supplementary Figure 5: Balance plot before and after conducting propensity score matched analysis on census tracts that did and did not reside in treated cities.**


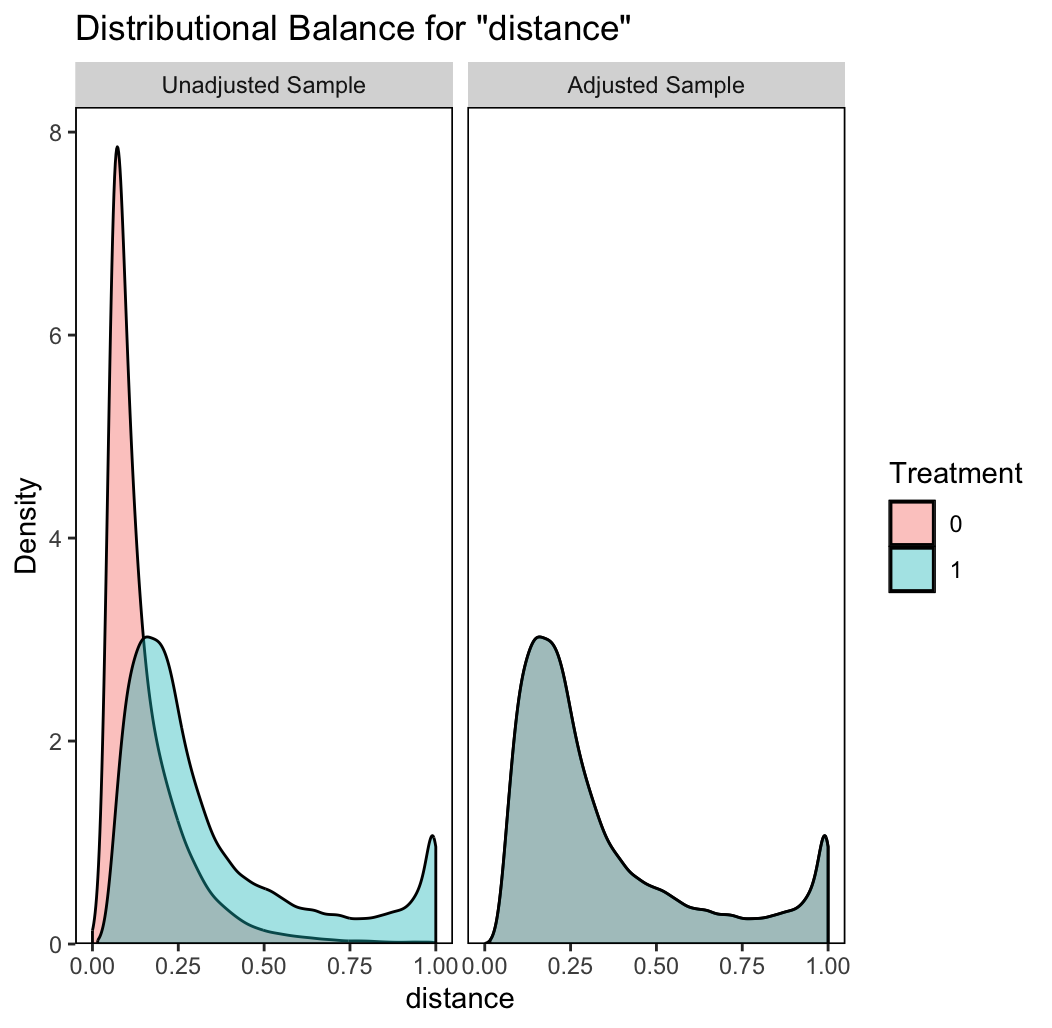


**Supplementary Figure 6: Covariate balance before and after matching.**


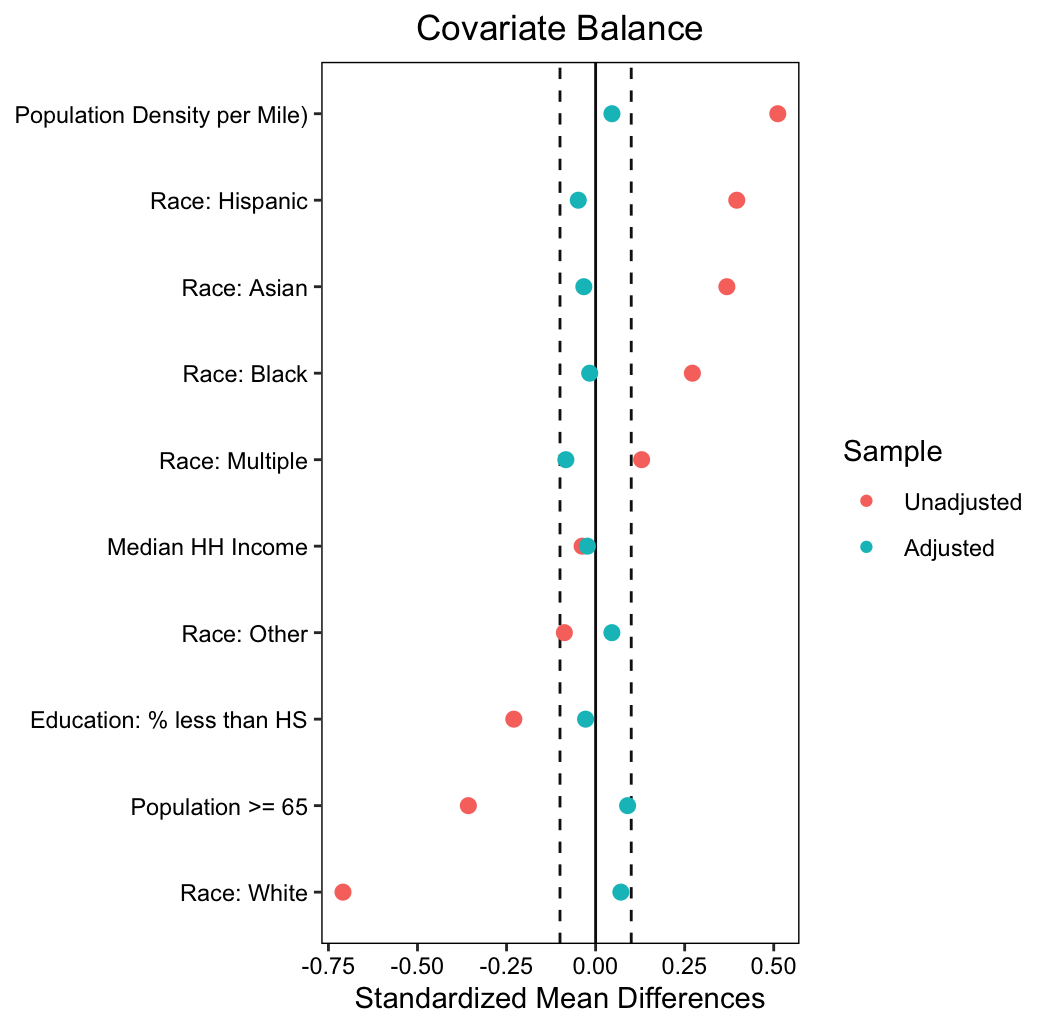

Supplement: 1 [file NIHMS2189782-supplement-1.docx]
